# Incidence of Diagnostic Errors Among Unexpectedly Hospitalized Patients Using an Automated Medical History–Taking System With a Differential Diagnosis Generator: Retrospective Observational Study

**DOI:** 10.2196/35225

**Published:** 2022-01-27

**Authors:** Ren Kawamura, Yukinori Harada, Shu Sugimoto, Yuichiro Nagase, Shinichi Katsukura, Taro Shimizu

**Affiliations:** 1 Department of Diagnostic and Generalist Medicine Dokkyo Medical University Mibu Japan; 2 Department of Internal Medicine Nagano Chuo Hospital Nagano Japan

**Keywords:** artificial intelligence, automated medical history–taking, diagnostic errors, outpatient, Safer Dx

## Abstract

**Background:**

Automated medical history–taking systems that generate differential diagnosis lists have been suggested to contribute to improved diagnostic accuracy. However, the effect of these systems on diagnostic errors in clinical practice remains unknown.

**Objective:**

This study aimed to assess the incidence of diagnostic errors in an outpatient department, where an artificial intelligence (AI)–driven automated medical history–taking system that generates differential diagnosis lists was implemented in clinical practice.

**Methods:**

We conducted a retrospective observational study using data from a community hospital in Japan. We included patients aged 20 years and older who used an AI-driven, automated medical history–taking system that generates differential diagnosis lists in the outpatient department of internal medicine for whom the index visit was between July 1, 2019, and June 30, 2020, followed by unplanned hospitalization within 14 days. The primary endpoint was the incidence of diagnostic errors, which were detected using the Revised Safer Dx Instrument by at least two independent reviewers. To evaluate the effect of differential diagnosis lists from the AI system on the incidence of diagnostic errors, we compared the incidence of these errors between a group where the AI system generated the final diagnosis in the differential diagnosis list and a group where the AI system did not generate the final diagnosis in the list; the Fisher exact test was used for comparison between these groups. For cases with confirmed diagnostic errors, further review was conducted to identify the contributing factors of these errors via discussion among three reviewers, using the Safer Dx Process Breakdown Supplement as a reference.

**Results:**

A total of 146 patients were analyzed. A final diagnosis was confirmed for 138 patients and was observed in the differential diagnosis list from the AI system for 69 patients. Diagnostic errors occurred in 16 out of 146 patients (11.0%, 95% CI 6.4%-17.2%). Although statistically insignificant, the incidence of diagnostic errors was lower in cases where the final diagnosis was included in the differential diagnosis list from the AI system than in cases where the final diagnosis was not included in the list (7.2% vs 15.9%, *P*=.18).

**Conclusions:**

The incidence of diagnostic errors among patients in the outpatient department of internal medicine who used an automated medical history–taking system that generates differential diagnosis lists seemed to be lower than the previously reported incidence of diagnostic errors. This result suggests that the implementation of an automated medical history–taking system that generates differential diagnosis lists could be beneficial for diagnostic safety in the outpatient department of internal medicine.

## Introduction

Diagnostic error, defined as the failure to establish an accurate and timely explanation of the patient’s health problem or to communicate that explanation to the patient [1], is one of the most important patient safety issues that should be addressed [2,3]. The impact of diagnostic errors on patient safety is quite large [4]. First, diagnostic errors comprise around 50% of preventable harm in primary health care settings and emergency departments [5]. Second, the risk of death, significant permanent injury, and prolonged hospitalization is higher for diagnostic error cases than for other medical errors [6-12]. Third, diagnostic errors frequently occur in several settings of clinical practice; approximately 5% of patients can experience diagnostic errors in primary health care and hospital practice in the United States [13]. Therefore, effective interventions to reduce diagnostic errors are warranted.

Diagnostic error–related paid malpractice claims occur more often among outpatients than among inpatients [9], suggesting that the primary health care outpatient setting is vulnerable to diagnostic errors. The prevalence of diagnostic errors in outpatient settings has been reported to be between 3.6% and 5.1%. However, when focusing on a population of patients with a high risk for diagnostic errors who were unexpectedly hospitalized within 14 days after the index outpatient visit, the prevalence of diagnostic errors increased to as much as 21% [14]. The common contributing factors for diagnostic errors in primary care outpatient settings were reported to include problems with history-taking, overreliance on pattern recognition, and failure to consider sufficient differential diagnoses [4,15]. Therefore, strategies or systems to improve the quality of history-taking and support differential diagnosis generation are required to reduce diagnostic errors in outpatient settings.

From this perspective, newly developed technology, such as computerized automated history-taking systems and diagnostic decision support systems, can be leveraged to address this issue; these systems have a long history, since they were introduced in the 1960s and 1970s [16-18]. Computerized automated history-taking systems perform better in clinical documentation tasks for taking patient histories than do physicians [19,20]. The use of a diagnostic support system (ie, differential diagnosis generator) before collecting information by physicians showed a significant impact on the improvement of diagnostic accuracy in terms of clinical reasoning and differential diagnosis [21-23]. Moreover, a new system that combines automated medical history–taking functions with differential diagnosis generation—specialized for musculoskeletal diseases only—showed improved diagnostic accuracy among physicians in a pilot randomized controlled trial [24]. Subsequently, another system, covering broad symptoms of internal diseases, was developed and implemented in clinical practice [25]. Yet another study showed high reliability of documentation regarding clinical history to assist the diagnostic accuracy of physicians [26]; however, this was not conducted in a clinical practice setting.

These automated systems have generated concerns about their negative effects on the diagnostic accuracy of physicians. For instance, physicians may not accept correct diagnoses or may accept incorrect diagnoses generated by the systems [24,26], partly because physicians tend to be more confident with their own diagnosis than that of artificial intelligence (AI) systems when there is a discrepancy between them [27]. Therefore, the effects of the implementation of these systems on diagnostic errors in clinical practice remain unknown. This study aimed to assess the incidence of diagnostic errors in an outpatient department, where an AI-driven automated medical history–taking system that generates differential diagnosis lists was implemented in clinical practice.

## Methods

### Study Design

We conducted a retrospective observational study using data from Nagano Chuo Hospital in Japan. The Research Ethics Committee of Nagano Chuo Hospital approved this study (serial number: NCR202104). The requirement to obtain written informed consent from patients was waived by the Research Ethics Committee under the condition that we used an opt-out method. We informed patients by showing the detailed information of the study on the official website of Nagano Chuo Hospital.

### Patient Population

We included patients aged 20 years and older who used AI Monshin—an AI-based automated medical history–taking system—in the outpatient department of internal medicine for whom the index visit was between July 1, 2019, and June 30, 2020, followed by unplanned hospitalization within 14 days. A follow-up duration of 14 days was selected to improve the sensitivity to detect diagnostic errors [14,28]. For assessing the effects of using AI Monshin on diagnostic errors, we excluded patients for whom AI Monshin did not list 10 differential diagnoses. In those cases, the AI system could not complete history-taking because patients gave up entering information or because they presented to the hospital for further investigation of abnormal test results following their annual health checkup, which was out of scope for the system during the study period. Usually, even one differential diagnosis was not generated in such cases.

### Presentation of the AI Monshin Tool

The details of AI Monshin were presented in a previous report [25]. In brief, AI Monshin converts data entered by patients on tablet terminals into medical terms. Patients enter their background information, such as age and sex, and chief complaint as free text on a tablet in the waiting room. AI Monshin asks approximately 20 questions, one by one, which are tailored to the patient. The questions are optimized, based on previous answers, to generate the most relevant list of potential differential diagnoses. Physicians can see the entered data as a summarized medical history with the top 10 possible differential diagnoses, along with their rank.

### Identification of Diagnostic Errors

To identify whether diagnostic errors occurred in this study, we used the Revised Safer Dx Instrument [29]. The Safer Dx Instrument is an externally validated, structured data collection tool to improve the accuracy of assessment of diagnostic errors [30,31]; the tool has been widely used in several studies on diagnostic errors [32-36]. Recently, the tool was updated as the Revised Safer Dx Instrument [29]. The Revised Safer Dx Instrument consists of 13 items. Items 1 to 12 are used for assessing the diagnostic process, and item 13 is used to determine the possibility of diagnostic error. All items are rated by answering questions on a scale ranging from 1 (strongly disagree) to 7 (strongly agree). The Revised Safer Dx Instrument can be used to assess the entire diagnostic process of one event; however, because we focused on diagnostic errors related to the implementation of AI Monshin, which seems to mainly influence the diagnostic decision at the index visit, the evaluation of diagnostic errors in this study was based on the medical records taken during the index visit.

The identification of diagnostic errors in this study was conducted through the algorithm as discussed in this section. In the first step, two reviewers (YH and SS) independently evaluated the diagnostic process of included cases using the Revised Safer Dx Instrument by reviewing the medical records. The presence or absence of diagnostic errors in each case was judged based on the score of item 13 [29]. According to the recommendation for using the Revised Safer Dx Instrument, diagnostic error was confirmed in cases where both reviewers scored 5 or higher on item 13, and diagnostic error was denied in cases where both reviewers scored 3 or lower on item 13 [29]. The remaining cases were progressed to the second step. In the second step, the third reviewer (YN) independently evaluated the cases using the Revised Safer Dx Instrument. Diagnostic error was confirmed in cases where two out of three reviewers scored 5 or higher on item 13, and diagnostic error was denied in cases where two out of three reviewers scored 3 or lower on item 13. For the remaining cases in which diagnostic error was neither confirmed nor denied, the three reviewers (YH, SS, and YN) discussed and mutually agreed on whether diagnostic error occurred or not on a case-by-case basis.

The final diagnoses of all cases were confirmed by two reviewers (YH and SS) based on the discharge summary. Disagreements were resolved by discussion among the three reviewers (YH, SS, and YN). Based on the confirmed final diagnoses, the other two reviewers (RK and SK), who were blinded to the evaluation of diagnostic errors, independently judged whether the final diagnosis of each case was included in the list of 10 differential diagnoses generated by AI Monshin. Disagreements were resolved by discussion between the two reviewers (RK and SK).

### Analysis of the Causes of Diagnostic Errors

For cases with confirmed diagnostic errors, further review was conducted to identify the contributing factors of these errors via discussion among the three reviewers (YH, SS, and YN). The Safer Dx Process Breakdown Supplement was used as a reference to classify the contributing factors of diagnostic errors and outcomes in this study [29]. To evaluate the effects of AI Monshin implementation on the diagnostic errors, other than the items in the Safer Dx Process Breakdown Supplement, the following were discussed: the frequency of the final diagnosis (ie, whether the disease was common or uncommon), typicality of the presentation for the final diagnosis (ie, typical or atypical), and initial diagnosis at the index visit.

### Baseline Data Collection and Outcome

From the medical records, we extracted data on the age and sex of patients, chief complaints, and the experience of physicians who saw patients at the index visits (ie, resident: up to 5 years of experience after graduation; staff: more than 5 years of experience after graduation). The primary outcome was the incidence of diagnostic errors.

### Sample Size Calculation

We calculated the required sample size to be 139 cases, with an incidence of diagnostic errors of 10.0% and a margin of 5.0%. It was estimated that there were approximately 150 patients who were eligible for this study between July 1, 2019, and June 30, 2020. Even with the expectation that approximately 5 to 10 cases could be excluded, 150 cases were a reasonable target number of cases for this study.

### Statistical Analysis

Continuous data are presented as medians with the 25th and 75th percentiles. Categorical data are presented as counts and proportions (%). For the primary outcome, we calculated the incidence of diagnostic errors with 95% CI. To evaluate the baseline factors and the differential diagnosis list of AI Monshin with regard to the incidence of diagnostic errors, we compared the incidence of diagnostic errors between the groups of older adults (aged ≥65 years) and non–older adults (aged <65 years) [37-40], the groups of males and females [33], the groups seen by staff and seen by residents [26], and the groups in which AI Monshin generated or did not generate the final diagnosis in the differential diagnosis list [26]; these comparisons were made using the Fisher exact test. We also calculated the odds ratio (OR) with 95% CI for the incidence of diagnostic errors in these groups. *P* values were based on 2-tailed statistical tests, and *P* values less than .05 were considered statistically significant. All statistical analyses were conducted using R (version 4.1.0; The R Foundation).

## Results

### Baseline Patient Characteristics

A total of 150 cases were unexpectedly hospitalized within 14 days after the index visit that took place at the outpatient department of internal medicine; AI Monshin was used at the index visit. Only 2 (1.3%) patients did not complete history-taking by AI Monshin: a woman in her 70s complained of an uncomfortable feeling on her tongue, abdominal pain with distention, and appetite loss, and a man in his 70s complained that his cold was not getting better. After excluding 4 (2.7%) cases in which AI Monshin did not develop 10 differential diagnoses (2 cases: incomplete history-taking; 2 cases: patients presented for further investigation for abnormal test results), the data from 146 cases were analyzed for this study. The median age of the patients was 71 (IQR 59-82) years, 72 (49.3%) were male, 71 (48.6%) were seen by residents at the index visit, and 103 (70.5%) were admitted to the hospital on the same day as the index visit.

### Chief Complaints and the Final Diagnosis

The top three most common chief complaints were abdominal pain (37/146, 25.3%), fever (20/146, 13.7%), and melena or hematochezia (15/146, 10.3%). During follow-up outpatient visits or admission, the final diagnosis was confirmed for 138 patients (94.5%). The most common diagnosis was lower respiratory tract infection (15/138, 10.9%), followed by ischemic colitis (8/138, 5.8%), diverticular bleeding (8/138, 5.8%), and congestive heart failure (8/138, 5.8%). The final diagnosis was based on the differential diagnosis list from AI Monshin for 69 out of 138 patients (50.0%).

### Primary Outcome

**Figure 1:**
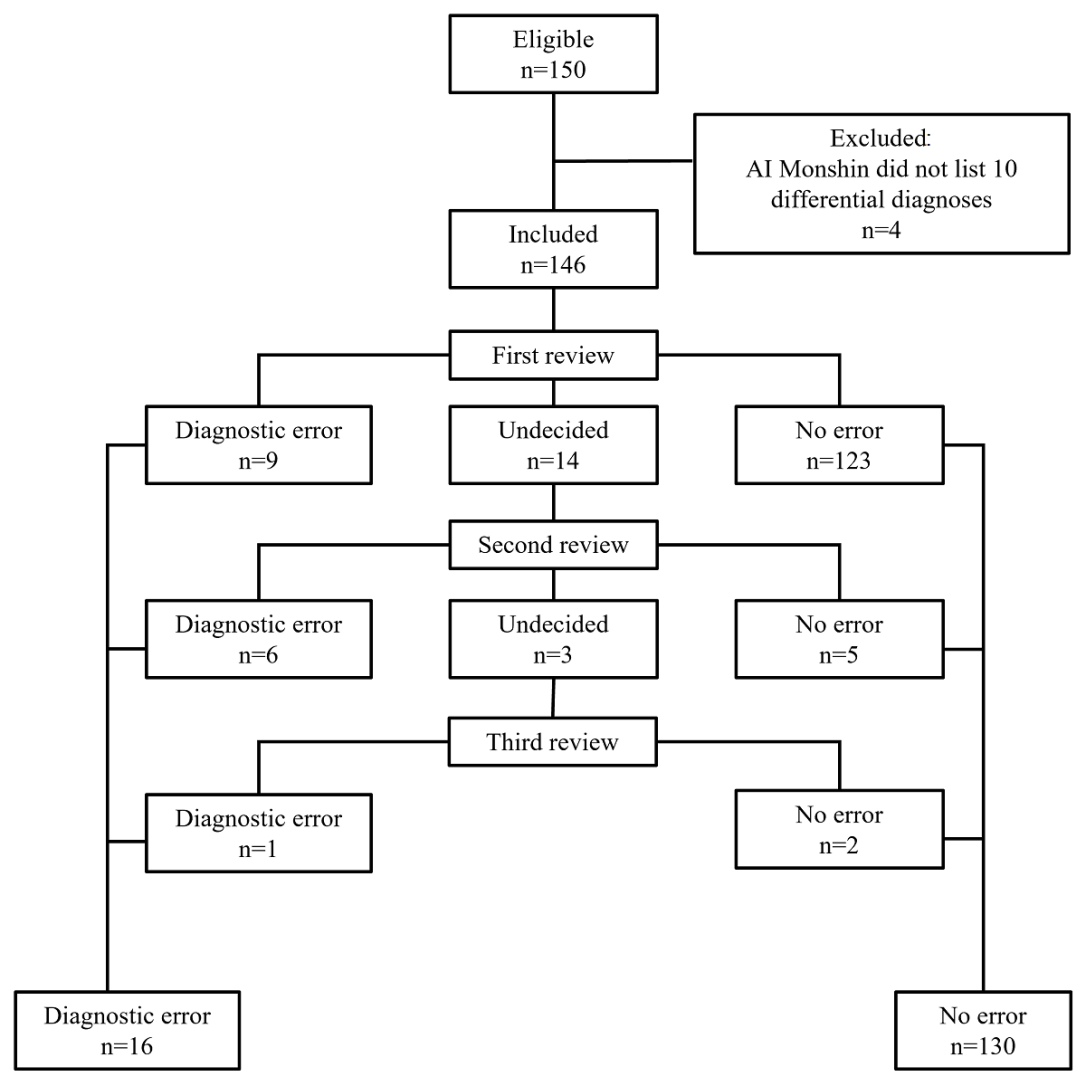
Flow of reviews for confirming diagnostic errors. AI: artificial intelligence.

Figure 1 shows the steps of the review for confirming the diagnostic errors in this study. In the first step of the review, diagnostic errors were confirmed in 9 cases and denied in 123 cases. Among the remaining 14 cases, diagnostic errors were confirmed in 6 cases and denied in 5 cases in the second step of the review. Among the remaining 3 cases, diagnostic errors were confirmed in 1 case and denied in 2 cases in the third step of the review. In total, diagnostic errors were confirmed in 16 out of 146 cases (11.0%, 95% CI 6.4%-17.2%).

The incidence of diagnostic errors was significantly higher in patients aged 65 years and older compared to those under 65 years of age (15/96, 16% vs 1/50, 2%; OR 9.1, 95% CI 1.2-70.8; *P*=.01). There were no significant differences in the incidence of diagnostic errors between male and female patients (11/72, 15% vs 5/74, 7%; OR 2.5, 95% CI 0.8-7.6; *P*=.12), between patients who were seen by a resident and those who were seen by a physician at the index visit (9/71, 13% vs 7/75, 9%; OR 1.4, 95% CI 0.5-4.0; *P*=.60), and between cases in which the final diagnosis was not included in the differential diagnosis list from AI Monshin and those in which the final diagnosis was included in the same list (11/69, 16% vs 5/69, 7%; OR 2.4, 95% CI 0.8-7.4; *P*=.18).

### Details Regarding Cases With Diagnostic Errors

Table 1 and Multimedia Appendix 1 show the details of the 16 cases where there were diagnostic errors. All cases had common final diagnoses (ie, cholangitis, cholecystitis, diverticular bleeding, pneumonia, interstitial pneumonia, intestinal obstruction, pyelonephritis, infectious enteritis, heart failure, and pulmonary artery embolism), and the final diagnosis presentation was typical for 15 out of 16 cases (94%). The most common chief complaint in the 16 cases with diagnostic errors was abdominal pain (n=5, 31%), followed by cough (n=4, 25%) and fever (n=3, 19%).

According to the Safer Dx Process Breakdown Supplement, the most common contributing factors for diagnostic errors in 16 cases were “problems ordering diagnostic tests for further workup” (n=13, 81%), followed by “problems with data integration and interpretation” (n=10, 63%), “problems with physical exam” (n=9, 56%), and “performed tests not interpreted correctly” (n=8, 50%; Table 2).

From the aspect of the differential diagnosis list for cases with diagnostic errors, AI Monshin listed the final diagnosis in the list in 5 out of 16 cases (31%) and the initial diagnosis in 4 out of 16 cases (25%). On the other hand, in cases without diagnostic errors, AI Monshin listed the final diagnosis in the differential list in 64 out of 122 cases (52.5%, excluding 8 cases where the final diagnosis was unknown). In summary, despite using AI Monshin, physicians could not make the correct diagnoses as were suggested in the differential diagnosis list in 5 of 69 cases (7% omission errors). On the other hand, the incorrect initial diagnoses made by physicians were listed in the differential diagnosis list in 4 of 69 cases (6% commission errors). Regarding the outcome, no cases of diagnostic errors resulted in death or permanent harm. A total of 2 cases out of 16 (13%) were classified as Category C: “An error occurred that reached the patient but did not cause the patient harm.” Diagnostic errors resulted in some harm in 14 out of 16 cases (88%; 2 cases were classified as Category E: “An error occurred that may have contributed to or resulted in temporary harm to the patient and required intervention”; 12 cases were classified as Category F: “An error occurred that may have contributed to or resulted in temporary harm to the patient and required initial or prolonged hospitalization”). The median time between the index visit and the time that the final diagnosis was made was 3 (IQR 2-6) days.

**Table 1:** The details of 16 diagnostic error cases.

Case No.^a^	Age (y)	Sex^b^	Physician of first contact	Chief complaint	Initial diagnosis	Final diagnosis	Index visit to final diagnosis (days), n	Outcome category^c^	Initial diagnosis was on list^d^	Final diagnosis was on list^d^
1	95	F	Resident	Fever	URI^e^	Cholangitis	4	F	No	No
2	76	M	Resident	Abdominal pain	GERD^f^	Cholecystitis	2	F	Yes; rank 4	No
3	83	M	Resident	Abdominal pain	Costochondritis	Pneumonia	3	F	No	No
4	55	M	Resident	Hematochezia	Infectious enteritis	Diverticular bleeding	2	F	Yes; rank 3	Yes; rank 1
5	89	F	Staff	Nausea	Unknown	Acute pyelonephritis	3	F	No	No
6	75	M	Staff	Cough	URI	Interstitial pneumonia	3	F	No	Yes; rank 10
7	66	M	Resident	Abdominal pain	Constipation	Intestinal obstruction	6	F	Yes; rank 4	No
8	70	F	Staff	Cough	Unknown	Heart failure	3	F	No	Yes; rank 8
9	77	F	Resident	Palpitation	Heart failure	Pulmonary embolism	2	E	Yes; rank 10	No
10	82	M	Staff	Fever	URI	Cholecystitis	3	F	No	No
11	81	F	Resident	Anorexia	Choledocholithiasis	Acute pyelonephritis	2	C	No	No
12	72	M	Staff	Headache, lightheadedness	Fatigue	Vestibular neuritis	8	E	No	No
13	86	M	Resident	Abdominal pain	Enteritis	Intestinal obstruction	0^g^	F	No	Yes; rank 9
14	78	M	Staff	Abdominal pain	Hemorrhoid	Infectious enteritis	9	C	No	No
15	91	M	Staff	Fever, cough, back pain	URI	Acute pyelonephritis	7	F	No	Yes; rank 3
16	72	M	Resident	Dyspnea, cough, malaise	URI	Interstitial pneumonia	11	F	No	No

^a^All diagnoses were common. All cases had typical presentations except for case 2.

^b^Female (F) or male (M).

^c^Outcome was classified, along with the Safer Dx Process Breakdown Supplement, as follows: Category C, “An error occurred that reached the patient but did not cause the patient harm”; Category E, “An error occurred that may have contributed to or resulted in temporary harm to the patient and required intervention”; Category F, “An error occurred that may have contributed to or resulted in temporary harm to the patient and required initial or prolonged hospitalization” [29].

^d^AI Monshin’s differential list; where a diagnosis was on the list, its rank on the list is indicated.

^e^URI: upper respiratory infection.

^f^GERD: gastroesophageal reflux disease.

^g^The final diagnosis was made at the second visit, which was on the same day as the index visit.

**Table 2:** Breakdown analysis of the contributing factors for diagnostic errors.

Contributing factors and details			Cases (N=16), n (%)
**Patient-related factors**			
	Delay in seeking care	0 (0)	
	Lack of adherence to appointments	0 (0)	
	Other	0 (0)	
**Patient-provider encounter**			
	Problems with history	4 (25)	
	Problems with physical exam	9 (56)	
	Problems ordering diagnostic tests for further workup	13 (81)	
	Failure to review previous documentation	4 (25)	
	Problems with data integration and interpretation	10 (63)	
	Other	0 (0)	
**Diagnostic tests**			
	Ordered test was not performed at all	0 (0)	
	Ordered test was not performed correctly	0 (0)	
	Performed test was not interpreted correctly	8 (50)	
	Misidentification	1 (6)	
	Other	0 (0)	
**Follow-Up and tracking**			
	Problems with timely follow-up of abnormal diagnostic test results	1 (6)	
	Problems with scheduling of appropriate and timely follow-up visits	2 (13)	
	Problems with diagnostic specialties returning test results to clinicians	2 (13)	
	Problems with clinicians reviewing test results	0 (0)	
	Problems with clinicians documenting action or response to test results	0 (0)	
	Problems with notifying patients of test results	0 (0)	
	Problems with monitoring patients through follow-up	0 (0)	
	Other	0 (0)	
**Referrals**			
	Problems initiating referral	1 (6)	
	Lack of appropriate actions on requested consultation	0 (0)	
	Communication breakdown from consultant to referring provider	0 (0)	
	Other	0 (0)	

## Discussion

### Principal Findings

Among 146 patients who used the AI-driven, automated history-taking system, which developed a list of the top 10 differential diagnoses, diagnostic errors occurred in 11.0% of cases. These patient histories were collected at the index visit to the outpatient department of internal medicine, followed by unplanned hospitalization of the patient within 14 days. The incidence of diagnostic errors was statistically higher among older adult patients; however, the sex of the patients, the experience of the physicians, and the accuracy of the differential diagnosis list of the AI system were not statistically associated with the incidence of diagnostic errors. In all cases where diagnostic errors occurred, the final diagnoses were common diseases, as reported in a previous study that was conducted in primary care settings in the United States between 2006 and 2007 [4], and the clinical presentation was typical, except in one case.

### Limitations

To the best of our knowledge, this is the first observational study that evaluated the effects of implementation of an automated medical history–taking system with a differential diagnosis generator in routine clinical practice using the validated Revised Safer Dx Instrument to detect diagnostic errors. However, this study also had some limitations. First, this study did not include patients who did not use an automated history-taking system with a differential diagnosis generator or those who were not admitted; therefore, the incidence of diagnostic errors should be interpreted with caution. Second, exclusion of the cases in which AI Monshin did not develop 10 differential diagnoses may have reduced the incidence of diagnostic errors in this study. Since inadequate and inappropriate history could be a contributing factor for diagnostic errors, excluding such a case may merit the optimistic assumption of AI Monshin’s performance. Third, because the judgment of diagnostic errors was conducted by a retrospective review of the charts, some bias could not be avoided. However, as the review process was predefined and at least two reviewers independently assessed each case, we are sure that these biases were avoided as much as possible. Fourth, we are unsure of the effects of COVID-19 on diagnostic errors in the outpatient department. Future studies may focus on the incidence of diagnostic errors between hospitals with and without implementation of an automated medical history–taking system with a diagnostic decision support function in a prospective design.

### Comparison With Prior Work

The incidence of diagnostic errors in this study was 11.0%, which was lower than that reported in previous studies (13.7% and 20.9%) that included cases similar to this study (ie, patients who were unexpectedly hospitalized within 14 days after their index visit) [14,28]. In addition, the incidence of diagnostic errors in this study was lower than that reported in retrospective studies with chart review (13.3% to 21.8%) [11,41-43] or in prospective studies (12.3% to 20.0%) [12,44] that investigated the rate of discrepancy in the diagnosis between admission and discharge. Therefore, it is possible that the implementation of an automated history-taking system with a differential diagnosis generator reduced the incidence of diagnostic errors in the outpatient department of internal medicine.

The quality of clinical history documented by AI Monshin may be a key component of the results. There may be high discrepancies in clinical history between patient reports and physician documentation [45]; in addition, the automated medical history–taking system, as compared to physicians, may have the potential to take clinical histories that are more diagnostically useful and of higher quality [19,20]. Therefore, routine use of automated history-taking systems may improve diagnostic accuracy by establishing a high-quality base of clinical history for the correct diagnosis. Indeed, in a previous study that used the documentation made by an automated medical history–taking system from real patients, the correct diagnosis appeared in 56.3% of the top three differential diagnoses made by physicians without using a differential diagnosis list from an AI-driven system; this increased to 72.7% in cases where the correct diagnosis was included in the AI-driven differential diagnosis list [26]. Furthermore, a previous study of another automated medical history–taking system with a differential diagnosis generator—DIAANA, specializing in injury or disease of the musculoskeletal system—showed that the diagnostic accuracy was superior in the group in which physicians used the system compared to the group in which physicians did not use the system; this was a pilot randomized controlled trial conducted in a real clinical practice setting [24]. In contrast to the previous study that identified history-taking as the most common contributing factor of diagnostic errors [4], the breakdown analysis of the diagnostic errors in this study did not identify history-taking as the main contributing factor of these errors, indicating that the implementation of an automated history-taking system with diagnostic decision support could reduce the diagnostic errors associated with poor clinical history–taking.

In addition to making a high-quality document of medical history, an automated medical history–taking system with a differential diagnosis generator seems to have some advantages. First, this system can be integrated into routine diagnostic processes in clinical practice. Currently, one of the most important concerns in the diagnostic decision support system is its low usage rate. For example, in the case of Isabel, which is one of the most famous AI-driven diagnostic decision support systems that generates a differential diagnosis list based on entered information by physicians, a previous study showed that only 7.9% of participants who were given open access to Isabel reported using Isabel at least once a week, whereas the others never used it [46]. According to the other two studies, on average, Isabel was used for only 3 out of 4840 patients (0.06%) for 3 months [47], and the usage rate did not increase despite frequent reminders for clinicians to use Isabel on a regular basis [48]. Such low use of a diagnostic decision support system appeared to be caused by physicians who did not recognize the need for diagnostic support, relying on their own acumen to deliver the correct diagnosis [49]. However, diagnostic decision support systems should operate seamlessly in the background in the diagnostic process in clinical practice, regardless of whether the physicians need it or not [49]. An automated medical history–taking system with a differential diagnosis generator can address such an unmet need and may reduce diagnostic errors through routine support. Second, the use of a diagnostic decision support system at the early stage of the diagnostic process was reported to be more useful than its use at a later stage. To date, several studies have been conducted to evaluate the impact of the timing of using a diagnostic decision support system. According to their studies, physician diagnosis was associated with their first impression [50], and early use of diagnostic support systems before collecting information by physicians significantly improved the diagnostic accuracy [21-23]. These findings may support the positive effects of the implementation of an automated medical history–taking system with a differential diagnosis generator, which can provide diagnostic decision support before physicians collect information. Third, an automated medical history–taking system with a differential diagnosis generator can be used without additional time consumption. Another barrier for clinicians to use diagnostic decision support systems in routine clinical practice is time constraint, as previous studies have shown that using Isabel usually requires an additional 4 to 7 minutes per case [47,48]. On the other hand, an automated history-taking system with a differential diagnosis generator increased only 0.3 minutes of examination time per case in an internal medicine outpatient department [25]. Therefore, clinicians can use automated history-taking systems with differential diagnosis generators without wasting additional time.

Furthermore, several limitations exist regarding the implementation of automated history-taking systems with differential diagnosis generators. First, at present, the accuracy of differential diagnosis lists of AI systems is not sufficiently high to believe the lists every time. A previous study reported that the prevalence of the correct diagnosis in the top 10 list of differential diagnoses from diagnostic decision support systems in clinical practice settings was around 50% [51]; similar to that study, the correct diagnosis appeared in only 50% of the top 10 lists of differential diagnoses from AI Monshin in this study. As an a priori incorrect diagnosis before a patient encounter can lead physicians to an incorrect final diagnosis [52], the relatively low accuracy of the differential diagnosis list from AI Monshin may prevent the positive effect of the implementation of an automated history-taking system with a differential diagnosis generator on the reduction of diagnostic errors. Although statistically insignificant, the incidence of diagnostic errors in cases where the correct diagnosis was included in the differential diagnosis list from the AI system was twice as high as that in cases where the correct diagnosis was not included in the list. However, among the 69 cases in which the final diagnosis was not included in the differential diagnosis list from the AI system, an incorrect diagnosis by a physician was observed in the differential diagnosis list from AI Monshin in only 4 cases (6%). In addition, a previous study showed that only 15% of physicians’ diagnoses seemed to be associated with the differential diagnosis list from the AI system [53]. This indicates that the majority of diagnostic errors in this study were not related to the incorrect differential diagnosis list from the AI system. Second, the correct diagnosis in the automated differential diagnosis list cannot always be accepted as the most likely diagnosis by a physician. In 5 out of 69 cases (7%) where the correct diagnosis was included in the AI-generated differential diagnosis list, the correct diagnosis was not accepted as the initial diagnosis by the physician in this study. However, this type of error was also lower than that reported in previous studies (10.0% and 15.9%) [24,53]. Third, automated medical history–taking systems have had difficulty in precise history-taking for specific patients, such as older adult patients [54]. Indeed, in cases with diagnostic errors in this study, important past medical history was not imputed for 3 patients. However, such missed information seemed to be easily covered by physicians by checking the past medical history directly from the patient or reviewing the previous documentation.

### Conclusions

The incidence of diagnostic errors seems to be reduced by the implementation of an automated medical history–taking system with a diagnostic decision support function in the outpatient department. Although the accuracy of the differential diagnosis list from AI Monshin remains low, the negative effects of incorrect differential diagnosis lists from AI systems on the diagnostic accuracy of physicians could be counteracted by the high-quality clinical history taken by AI systems. Therefore, in total, the implementation of an automated history-taking system with diagnostic decision support may have more beneficial impacts than negative effects on diagnostic safety in the outpatient department.

## Appendix

Multimedia Appendix 1 Details of the histories written by AI Monshin in 16 diagnostic error cases. AI: artificial intelligence.

## Abbreviations

AI: artificial intelligence
KAKENHI: Grants-in-Aid for Scientific Research
OR: odds ratio
