# Supplementary material for: Incidence of Diagnostic Errors Among Unexpectedly Hospitalized Patients Using an Automated Medical History–Taking System With a Differential Diagnosis Generator: Retrospective Observational Study
Source: JMIR Med Inform. 2022 Jan 27;10(1):e35225. doi: 10.2196/35225 (PMC8832260; doi:10.2196/35225)
Supplement: Multimedia Appendix 1 [file medinform_v10i1e35225_app1.docx]

Multimedia Appendix 1. The details of history written by artificial intelligence in 16 cases of diagnostic errors.

| No. | Clinical history taken by the automated medical history taking system | AI diagnosis list | Physician diagnosis list |
| --- | --- | --- | --- |
|  |  |  |  |
| 1 | [Age and Sex]  95-years-old woman  [Chief complaints]  Fever  [History of present illness]  Fever of 38℃ or higher appeared and continued without getting better or worse.  [Review of symptoms]  Positive: Edema, warmness in the limbs  Negative: Nausea, vomiting  [Past medical history]  Positive: Urinary tract infection  Negative: Hypertension, dyslipidemia, diabetes mellitus, coronary artery disease, cerebrovascular disease, malignancy  Allergy: None  OBGYN: 0G0P  [Social history]  Alcohol: None  Smoking: Never smoker  Other: No travel history within 1 month | 1. Osteomyelitis  2. Rheumatic fever  3. Cellulitis/Erysipelas  4. Chronic pyelonephritis  5. Mycoplasma infection  6. Acute pyelonephritis  7. Pneumonia (lower respiratory tract infection)  8. Infective endocarditis  9. Pneumothorax  10. Hypersensitivity pneumonitis | 1. Upper respiratory infection |
| 2 | [Age and Sex]  76-years-old man  [Chief complaints]  Abdominal pain  [History of present illness]  The patient developed heartburn 8 hours ago, which lasted for more than 30 minutes, and is getting better now. However, the symptoms are still present. The heartburn was a burning sensation in the epigastrium. The patient has never experienced the symptom before.  [Review of symptoms]  Positive: Obesity, snoring, sleep apnea  Negative: Nausea, vomiting, dysphagia, bloody stools, loss of appetite, chest pain, back pain, burping, dyspnea, headache, malaise, feeling to faint, cold sweat, palpitation  [Past medical history]  Positive: Hypertension, dyslipidemia, diabetes mellitus, abnormal finding in gastroscopy within one-year, gastroesophageal reflux disease  Negative: Coronary artery disease, cerebrovascular disease, malignancy  Allergy: None  Medications: Drugs for hypertension, dyslipidemia, and diabetes (details unknown)  [Social history]  Alcohol: 2 drinks of Japanese Sake twice a week  Smoking: Never smoker | 1. Sleep apnea syndrome  2. Gastric/Duodenal ulcer  3. Esophageal spasm (including achalasia and Jackhammer esophagus)  4. Reflux esophagitis  5. Functional dyspepsia  6. Aerophagia  7. Gastric cancer  8. Stable angina  9. Gastritis  10. Myocardial infarction/Unstable angina | 1. Gastroesophageal reflux disease  2. Acute coronary syndrome |
| 3 | [Age and Sex]  83-years-old man  [Chief complaints]  Abdominal pain  [History of present illness]  The patient developed sudden onset of pain in the right upper abdomen four days prior to the visit. The pain is intermittent, NRS 8/10, and has continued without getting better or worse. The patient has never experienced the symptom before. The patient also developed anorexia on the same day and developed constipation, nausea, and vomiting one day prior to the visit.  [Review of symptoms]  Positive: Chest pain (lasting less than 15 minutes), dyspnea (aggravated by exertion)  Negative: Diarrhea, bloody stool  [Past medical history]  Positive: Prostate cancer, appendicitis (appendectomy), hypertension, diabetes mellitus, other diseases (details unknown)  Negative: Dyslipidemia, coronary artery disease, cerebrovascular disease, malignancy  Allergy: positive for food (details unknown) and a drug (over-the-counter painkiller)  Medications: Tamsulosin 0.2 mg, Bicalutamide 80mg  [Social history]  Alcohol: None  Smoking: Current smoker (15 cigarettes per day from age 15) | 1. Gastric/Duodenal ulcer  2. Stable angina  3. Diabetic ketoacidosis  4. Myocardial Infarction/Unstable angina  5. Chronic hepatitis  6. Gastritis  7. Inguinal hernia  8. Acute cholecystitis  9. Aortic dissection/Ruptured aortic aneurysm  10. Acute pyelonephritis | 1. Costochondritis |
| 4 | [Age and Sex]  55-years-old man  [Chief complaints]  Hematochezia  [History of present illness]  The patient vomited 3- or 4-times last night and developed bloody diarrhea. In today’s morning, bloody diarrhea occurred twice.  [Review of symptoms]  Positive: Heartburn  Negative: Abdominal pain  [Past medical history]  Positive: Hypertension, coronary artery disease, other diseases (details unknown), abnormal finding in gastroscopy within one-year, peptic ulcer disease  Negative: Dyslipidemia, diabetes, cerebrovascular disease, malignancy  Allergy: None  Medications: Details unknown  [Social history]  Alcohol: None  Smoking: Former smoker (20 cigarettes per day from age 20 to 54)  Other: No travel history within 1 month | 1. Diverticular hemorrhage  2. Ischemic enteritis  3. Bacterial gastroenteritis  4. Ulcerative colitis  5. Piles/Anal fissure  6. Colorectal cancer  7. Gastric/Duodenal ulcer  8. Viral gastroenteritis  9. Gastric cancer  10. IgA Vasculitis (Schonlein-Henoch Purpura) | 1. Infectious enteritis |
| 5 | [Age and Sex]  89-years-old woman  [Chief complaints]  Nausea  [History of present illness]  The patient developed nausea and vomiting once or twice a day one day prior to the visit, which is getting better now, but the symptoms are still present. Nausea and vomiting worsen after eating, and nausea improves with vomiting. On the same day, a headache of NRS 3/10 appeared, which lasted from a few seconds to a few minutes. On the same day, abdominal pain of NRS 5/10 appeared, which improved after vomiting. On the same day, anorexia appeared to the extent that he could not eat but could drink water. The patient denies any sick contact.  [Review of symptoms]  Positive: Forgetfulness  Negative: Diarrhea  [Past medical history]  Positive: Carpal tunnel fracture, hip joint fracture, dyslipidemia, other diseases (details unknown)  Negative: Hypertension, diabetes mellitus, coronary artery disease, cerebrovascular disease, malignancy  Allergy: None  OBGYN: The patient has experience of childbirth (details unknown) and no experience of miscarriage or abortion.  Medications: Amlodipine 10 mg, Valsartan 160 mg, Lansoprazole 15 mg, Sodium Gualenate Hydrate, Simvastatin 5 mg  [Social history]  Alcohol: None  Smoking: Never smoker | 1. Cerebral infarction  2. Intestinal obstruction  3. Subarachnoid hemorrhage  4. Gastric/Duodenal ulcer  5. Diabetic ketoacidosis  6. Acute cholecystitis  7. Gastric cancer  8. Congestive heart failure  9. Cerebral hemorrhage  10. Chronic subdural hematoma | No differential diagnosis was written |
| 6 | [Age and Sex]  75-years-old man  [Chief complaints]  Cough  [History of present illness]  The patient developed a productive cough several times a day a month ago, and the cough has been getting worse. The cough is like a reprise and worsens with exercise. The patient has never experienced the symptom before. The patient also has nasal discharge and dyspnea. The patient sometimes wakes up at midnight due to the cough and dyspnea. The patient denies any sick contact.  [Review of symptoms]  None  [Past medical history]  Positive: Diabetes mellitus, coronary artery disease  Negative: Hypertension, dyslipidemia, cerebrovascular disease, malignancy  Allergy: None  [Social history]  Alcohol: None  Smoking: Former smoker (20 cigarettes per day from age 19 to 73) | 1. Asthma  2. Heart failure  3. Chronic obstructive pulmonary disease  4. Sinusitis  5. Pneumonia (lower respiratory tract infection)  6. Lung cancer  7. Hypersensitivity pneumonitis  8. Pulmonary fibrosis  9. Cough variant asthma  10. Upper airway cough syndrome | 1. Upper respiratory infection |
| 7 | [Age and Sex]  66-years-old man  [Chief complaints]  Abdominal pain  [History of present illness]  The patient developed sudden onset of pain in the epigastrium and lower abdomen 5 days ago, which has continued without getting better or worse. The degree of the pain is NRS 7/10. The pain is intermittent and worsens after eating oily foods. The patient also developed constipation three days ago.  [Review of symptoms]  Positive: Heartburn, abdominal distention  Negative: Nausea, vomiting, diarrhea, bloody stool, chest pain, back pain  [Past medical history]  Positive: None  Negative: Hypertension, dyslipidemia, diabetes mellitus, coronary artery disease, cerebrovascular disease, malignancy, gallstones, pancreatitis, peptic ulcer disease, urolithiasis  Allergy: None  Medications: Over-the-counter medication [details unknown]  [Social history]  Alcohol: Yes (details unknown)  Smoking: Yes (details unknown)  [Family history]  No colorectal cancer in the family | 1. Gastric/Duodenal ulcer  2. Gastric cancer  3. Colorectal cancer  4. Constipation  5. Functional dyspepsia  6. Inguinal hernia  7. irritable bowel syndrome  8. Acute pyelonephritis  9. Chronic Hepatitis  10. Diabetic ketoacidosis | 1. Constipation  2. Colorectal cancer |
| 8 | [Age and Sex]  70-years-old woman  [Chief complaints]  Cough  [History of present illness]  The patient developed a productive cough several times a day 10 days prior to the visit, and the cough has been getting worse. The cough is aggravated by exercise. The patient also developed dyspnea and heartburn. The cough and dyspnea worsen from night to dawn. The patient denies any sick contact.  [Review of symptoms]  None  [Past medical history]  Positive: Other disease (details unknown)  Negative: Hypertension, dyslipidemia, diabetes mellitus, coronary artery disease, cerebrovascular disease, malignancy  Allergy: Positive for a food (buckwheat)  OBGYN: 0G0P  Medications: None  [Social history]  Alcohol: None  Smoking: Never smoker | 1. Asthma  2. Chronic obstructive pulmonary disease  3. Pulmonary fibrosis  4. Pneumonia (lower respiratory tract infection)  5. Empyema  6. Hypersensitivity pneumonitis  7. Pneumothorax  8. Heart failure  9. Pulmonary embolism  10. Lung cancer | No differential diagnosis was written |
| 9 | [Age and Sex]  77-years-old woman  [Chief complaints]  Palpitation  [History of present illness]  The patient developed palpitation 4 days ago. On the same day, the patient developed edema in the right hand and periorbital area and dyspnea on exertion. The patient also developed a feeling of tension in the right shoulder 2 days ago.  [Review of symptoms]  Positive: Cold sweat, feeling a loss of consciousness, feeling upset or fearful.  Negative: Numbness, sensory disturbance, chest pain, dizziness  [Past medical history]  Positive: Hypertension, dyslipidemia  Negative: Diabetes mellitus, coronary artery disease, cerebrovascular disease, malignancy  Allergy: Positive for food (details unknown)  OBGYN: The patient has an experience of miscarriage or abortion (details unknown)  Medications: Tocopherol Acetate, Valsartan, Ezetimibe, Pravastatin, Carvedilol  [Social history]  Alcohol: None  Smoking: Never smoker | 1. Stable angina  2. Hyperventilation syndrome/Anxiety disorder  3. Psychosomatic disorder  4. Aortic stenosis  5. Myocardial infarction/Unstable angina  6. Anemia  7. Atrioventricular block/Sick sinus syndrome  8. Benign arrhythmias  9. Hypoglycemia  10. Heart failure | 1. Heart failure  2. Pulmonary embolism |
| 10 | [Age and Sex]  82-years-old man  [Chief complaints]  Fever  [History of present illness]  The patient developed a fever of 38℃ or higher one day prior to the visit and the fever has continued without getting better or worse. The patient also has pain around the right ribs. The patient denies any sick contact.  [Review of symptoms]  Positive: None  Negative: Dyspnea, abdominal pain, headache, gross hematuria  [Past medical history]  Positive: Hypertension, other diseases (details unknown)  Negative: Dyslipidemia, diabetes mellitus, coronary artery disease, cerebrovascular disease, malignancy  Allergy: None  Medications: Amlodipine 5mg, Aspirin 100mg, Lansoprazole 15mg  [Social history]  Alcohol: Half a glass of Shochu with hot water every day  Smoking: Never smoker  Other: No travel history within 1 month | 1. Acute pyelonephritis  2. Drug eruption/Toxic eruption/Drug fever  3. Empyema  4. Infective endocarditis  5. Pneumonia (lower respiratory tract infection)  6. Otitis media  7. Chronic pyelonephritis  8. Acute prostatitis  9. Cellulitis/Erysipelas  10. Hypersensitivity pneumonitis | 1. Upper respiratory infection |
| 11 | [Age and Sex]  81-years-old woman  [Chief complaints]  Anorexia  [History of present illness]  The patient developed depression around a month ago. Six days prior to the visit, the patient developed constipation. Five days prior to the visit, fever and anorexia appeared, and anorexia worsened. The patient also developed nausea and vomiting three days ago.  [Review of symptoms]  Positive: Arthralgia, increased frequency of urination, weakness, decreased activity  Negative: Abdominal pain, diarrhea, bloody stool, headache, dizziness  [Past medical history]  Positive: Hypertension, dyslipidemia  Negative: Diabetes mellitus, coronary artery disease, malignancy, cerebrovascular disease  Allergy: Positive for food and drug (details unknown)  OBGYN: The patient has experience of childbirth but has no experience of miscarriage or abortion (details unknown)  Medications: Medicines for digestion, Herbal medicines  [Social history]  Alcohol: None  Smoking: Current smoker (10 cigarettes per day from age 20) | 1. Cerebral infarction  2. Cerebral hemorrhage  3. Chronic subdural hematoma  4. Subarachnoid hemorrhage  5. Depression  6. Heart failure  7. Gastric/Duodenal ulcer  8. Malignant lymphoma  9. Myocardial infarction/Unstable angina  10. Parkinson's disease | 1. Choledocholithiasis  2. Acute pyelonephritis |
| 12 | [Age and Sex]  72-years-old man  [Chief complaints]  Headache, lightheadedness  [History of present illness]  The patient developed lightheadedness 10 days ago, which continued without getting better or worse. On the same day, the patient also developed a headache.  [Review of symptoms]  Positive: None  Negative: Chest pain, numbness, sensory disturbance  [Past medical history]  Positive: Hypertension, dyslipidemia, diabetes mellitus  Negative: Coronary artery disease, cerebrovascular disease, malignancy  Allergy: None  Medications: The patient took some medicines regularly, but details were unknown  [Social history]  Alcohol: Occasionally drinking  Smoking: Never smoker | 1. Cerebral hemorrhage  2. Chronic subdural hematoma  3. Aortic dissection/Ruptured aortic aneurysm  4. Hyperthyroidism  5. Electrolyte abnormalities  6. Cerebral infarction  7. Tic disorder  8. Parkinson's disease  9. Viral acute encephalopathy  10. Meningitis | 1. Fatigue  2. Meniere’s disease |
| 13 | [Age and Sex]  86-years-old man  [Chief complaints]  Abdominal pain  [History of present illness]  The patient developed sudden onset of pain in the left lower abdomen one day prior to the visit, which continued without getting better or worse. The pain was intermittent and worsened after eating or drinking and improved after defecation. The patient has never experienced the symptom before. The patient also developed anorexia on the same day. The patient remembered eating raw oysters, sashimi, old rice, undercooled liver within a week.  [Review of symptoms]  Positive: Oliguria  Negative: Nausea, vomiting, diarrhea, bloody stool  [Past medical history]  Positive: Hypertension, dyslipidemia, gastric cancer (gastrectomy, 81 years old), heart failure, intestinal obstruction (surgically treated, 84 years old), pacemaker implantation (details unknown, 84 years old)  Negative: Diabetes mellitus, coronary artery disease, cerebrovascular disease  Allergy: Positive for food (sardine) and metal (details unknown)  Medications: Amlodipine 2.5 mg, Brotizolam 0.50 mg, Butyl scopolamine 10mg, Minodronate 50 mg, Bepotastine 20 mg, Silodosin 8 mg  [Social history]  Alcohol: Shochu 50ml per day  Smoking: Former smoker (70 cigarettes per day from age 35 to 45) | 1. Aortic dissection/Ruptured aortic aneurysm  2. Myocardial infarction/Unstable angina  3. Gastric/Duodenal ulcer  4. Chronic hepatitis  5. Gastric cancer  6. Acute cholecystitis  7. Diabetic ketoacidosis  8. Acute cholangitis  9. Intestinal obstruction  10. intestinal perforation | 1. Enteritis  2. Intestinal obstruction |
| 14 | [Age and Sex]  78-years-old man  [Chief complaints]  Abdominal pain  [History of present illness]  The patient developed subacute onset pain in the lower abdomen seven days prior to the visit, which has been getting worse. The degree of pain is NRS 4/10. The pain is intermittent, lasting about 30 minutes or less, and improves after defecation. The patient has never experienced the symptom. Diarrhea occurred 5 days prior to the visit, and the patient noted bloody stools one day prior to the visit.  [Review of symptoms]  Positive: Narrowing of stool, awakening midnight due to diarrhea  Negative: Nausea, vomiting  [Past medical history]  Positive: Hypertension  Negative: Dyslipidemia, diabetes mellitus, coronary artery disease, cerebrovascular disease, malignancy  Allergy: None  Medications: Inderal and other medications for hypertension (details unknown)  [Social history]  Alcohol: None  Smoking: Current smoker (10 cigarettes per day from age 20) | 1. Irritable bowel syndrome  2. Colorectal cancer  3. Ulcerative colitis  4. Stable angina  5. Diverticulum bleeding  6. Gastric cancer  7. Acute pyelonephritis  8. Chronic hepatitis  9. Gastric/Duodenal ulcer  10. Urolithiasis | 1. Hemorrhoid  2. Colorectal cancer |
| 15 | [Age and Sex]  91-years-old man  [Chief complaints]  Fever, cough, back pain  [History of present illness]  The patient developed a fever of 37℃ range and back pain one day prior to the visit. The patient also developed a cough several times a day.  [Review of symptoms]  Positive: Peripheral coldness  Negative: Chest pain, nausea, vomiting  [Past medical history]  Positive: Hypertension, coronary artery disease, heart failure, other diseases (details unknown)  Negative: Diabetes mellitus, dyslipidemia, malignancy, cerebrovascular disease  Allergy: None  Medications: Clobetasol ointment, Betamethasone ointment, Hydrocortisone ointment, Bifonazole cream, Prostandin ointment, Rivaroxaban 10 mg, Bisoprolol 0.625 mg, Furosemide 20 mg, Spironolactone 25 mg, Bepotastine 10mg, Mequitazine 3 mg, Hydroxyzine 25mg  [Social history]  Alcohol: None  Smoking: Never smoker  Other: No travel history within 1 month | 1. Aortic dissection/Ruptured aortic aneurysm  2. Myocardial infarction/Unstable angina  3. Acute pyelonephritis  4. Malignant lymphoma  5. Hyperthyroidism  6. Drug eruption/Toxin eruption/Drug fever  7. Chronic pyelonephritis  8. Hypersensitive pneumonitis  9. Scleroderma  10. Empyema | 1. Upper respiratory infection |
| 16 | [Age and Sex]  72-years-old man  [Chief complaints]  Dyspnea, cough, malaise  [History of present illness]  The patient developed gradual onset of dyspnea a month ago, which is getting better but remains. The dyspnea lasts about 15 minutes or less and worsens with exertion. At the same time, the patient has also developed a cough several times a day. The patient had similar symptoms more than once before.  [Review of symptoms]  Positive: Malaise  Negative: Chest pain  [Past medical history]  Positive: None  Negative: Hypertension, dyslipidemia, diabetes mellitus, coronary artery disease, cerebrovascular disease, malignancy  Allergy: None  [Social history]  Alcohol: Yes (details unknown)  Smoking history: Never smoker | 1. Chronic obstructive pulmonary disease  2. Myasthenia gravis  3. Pneumonia (lower respiratory tract infection)  4. Anemia  5. Benign arrhythmias  6. Lung cancer  7. Atrioventricular block/Sick sinus syndrome  8. Psychosomatic disorder  9. Heart failure  10. aortic stenosis | 1. Upper respiratory infection |
